# Phenotypic and genotypic characterization of *Mycobacterium tuberculosis* pyrazinamide resistance—India, 2018–2020

**DOI:** 10.3389/fmicb.2024.1515627

**Published:** 2025-01-08

**Authors:** Sembulingam Tamilzhalagan, Evanslin Santus Justin, Ashok Selvaraj, Karthick Venkateswaran, Arun Kumar Sivakumar, Suganthi Chittibabu, Heather P. McLaughlin, Patrick K. Moonan, Jonathan P. Smith, Sakthi Suba, Mukesh Kumar Sathya Narayanan, Christine S. Ho, Nishant Kumar, Srikanth P. Tripathy, Siva K. Shanmugam, Patricia J. Hall-Eidson, Uma Devi Ranganathan

**Affiliations:** ^1^National Institute for Research in Tuberculosis, Indian Council of Medical Research, Chennai, India; ^2^Division of Global HIV and Tuberculosis, U.S. Centers for Disease Control and Prevention, Atlanta, GA, United States; ^3^Government of India, Ministry of Health and Family Welfare, National Tuberculosis Elimination Programme, New Delhi, India; ^4^Dr. D Y Patil Medical College, Hospital and Research Center, Dr. D Y Patil Vidyapeeth (Deemed to be University), Pimpri, Pune, India

**Keywords:** tuberculosis, drug resistance, drug susceptibility testing, genetic mutations, whole genome sequencing, pyrazinamide, PZA

## Abstract

Pyrazinamide (PZA) is a key first-line antituberculosis drug that plays an important role in eradicating persister *Mycobacterium tuberculosis* (TB) bacilli and shortening the duration of tuberculosis treatment. However, PZA-resistance is on the rise, particularly among persons with multidrug-resistant (MDR) tuberculosis. This nationwide study was conducted to explore the prevalence of mutations conferring PZA resistance, catalogue mutation diversity, investigate the associations of PZA resistance with specific lineages, examine co-resistance to 13 first- and second-line drugs, and evaluate the diagnostic accuracy of sequencing *pnc*A and *pan*D genes for predicting PZA resistance. Whole genome sequencing was performed on 2,207 *M. tuberculosis* isolates from 25 States and 4 Union Territories of India. The majority of phenotypically PZA-resistant isolates (77%) harbored 171 distinct mutations in *pnc*A; however, a small number of mutations in *pan*D, *rps*A and *clp*C1 were also observed. A set of novel mutations associated PZA resistance was uncovered, along with an additional 143 PZA resistance-conferring mutations in *pnc*A based on application of WHO-endorsed grading rules. PZA resistance was predominately observed in Lineage 2 and eight lineage-specific resistance markers were identified. Mutations distributed across *pnc*A correlate to 94% of PZA resistance and were the predominant drivers of phenotypic resistance; evidence generated herein substantiates sequencing the entire gene and promoter for comprehensive genotypic-based prediction of PZA resistance. This work provides key insights into the scope of PZA-resistance in India, a high drug-resistant TB burden country, and can support the effectiveness of TB prevention and control efforts.

## Introduction

In 2022, India reported the highest tuberculosis (TB) burden in the world. With an estimated 2,950,000 persons suffering from TB, including 119,000 persons with drug-resistant (DR) TB, India accounted for over a quarter of all TB cases, and 13% of all DR TB cases globally (Government of India Ministry of Health and Family Welfare, 2022). Standard treatment for pulmonary TB includes isoniazid (INH), rifampicin (RIF), ethambutol (EMB) and pyrazinamide (PZA). PZA contributes bactericidal activity against slow-growing and non-replicating populations of *Mycobacterium tuberculosis* (MTB) bacilli, acts synergistically with RIF to support treatment completion in 6 months (Heifets and Lindholm-Levy, 1992; Zhang and Mitchison, 2003), and reduces risk of clinical relapse (Mitchison, 1985). Recent research shows that PZA enters Mycobacterium tuberculosis by passive diffusion, is converted to pyrazinoic acid by nicotinamidase/pyrazinamidase (PZase), and accumulates under acidic conditions, causing cellular damage (Zhang and Mitchison, 2003). PZA is an essential component of drug-susceptible TB treatment (World Health Organization, 2022) and remains indispensable for DR TB treatment by shortening treatment periods and addressing persister mycobacteria populations (Fofana et al., 2024). Resistance to PZA can lead to unsuccessful treatment outcomes and acquisition of further resistance to other antituberculosis medicines (Whitfield et al., 2015). In 2018, the Indian national TB drug-resistance survey reported the overall resistance to PZA was 7% (*n* = 215) and 9% (*n* = 170) among all new and previously treated persons with TB, respectively and was higher 31% (*n* = 27) among persons with newly confirmed multidrug-resistant TB (MDR TB; defined as phenotypic resistance to INH and RIF) (Government of India Ministry of Health and Family Welfare, 2018).

To be effective against MTB, PZA must be hydrolyzed to pyrazinoic acid (POA) by the pyrazinamidase (PZAse) enzyme, which is encoded by the *pnc*A gene (Zhang and Mitchison, 2003; Miotto et al., 2014). Mutations across the promoter and coding regions of *pnc*A lead to reduced or lost PZase activity, which in turn results in PZA resistance (PZA^R^). Globally, over 80% of PZA-resistant isolates harbor single nucleotide, multi-nucleotide, insertion-based, or deletion-based mutations throughout the highly permissive regulatory and coding regions of the *pnc*A gene (Miotto et al., 2014). In addition, several other genes have been reported as harboring potential or confirmed PZA^R^-associated mutations (Rajendran and Palaniyandi, 2022; Ramirez-Busby and Valafar, 2015), albeit without confirmation of resistance association by the latest global dataset and mutations catalogue released by the World Health Organization (2023). These include *rps*A, which encodes the ribosomal protein S1 (Yang et al., 2015), *pan*D, which encodes an aspartate 1-decarboxylase required for coenzyme-A biosynthesis (Zhang et al., 2013), and more recently, *clp*C1, an ATP-dependent ATPase involved in protein degradation that may be associated with lineage-specific low-level PZA^R^ (Modlin et al., 2021). However, despite these known and potential molecular mechanisms of PZA^R^, the absence of a *pnc*A resistance-associated mutation “*hot spot*,” and lack of clear evidence for resistance association of the other reported genes, have hindered the design of rapid, targeted molecular drug susceptibility testing (DST) assays beyond a single high-complexity and heavily-infrastructure dependent option, leading to widespread continued reliance on culture-based phenotypic DST (pDST) for detection of PZA^R^ (Georghiou et al., 2023; Alcántara et al., 2020).

In India, capacity for molecular and pDST is being scaled rapidly to inform appropriate treatment selection and enhance disease control efforts through strengthened surveillance. Phenotypic DST for PZA is performed for strains isolated from persons presumed to have MDR TB, including those that were previously treated with a PZA-containing regimen without achieving cure (Government of India Ministry of Health and Family Welfare, 2021; Gupta et al., 2022). The current pDST method for PZA relies upon the BACTEC Mycobacterial Growth Indicator Tube (MGIT) 960 system (Becton Dickinson, Franklin Lakes, NJ, United States) which requires use of larger inoculum of MTB in a modified, acidic, MGIT culture media to ensure PZA activity can overcome the growth-limiting acidic conditions. Robust follow-on retesting is often necessary to rule out errors and false-positive resistance results (Chedore et al., 2010; Mok et al., 2021). In addition, the accuracy and turnaround time challenges limit the value of pDST results to guide inclusion or exclusion of PZA in treatment regimens, leading to prolonged treatment with PZA prior to resistance detection and reporting, elevating risk for acquired PZA^R^ and continued transmission (Zhang et al., 2012; Li et al., 2021).

In India, where the TB burden is high and PZA is routinely used in first-and second-line TB treatment regimens, understanding the scope and molecular epidemiology of PZA^R^ is pivotal for determination of its prevalence, diversity, and geographic distribution to inform targeted disease control efforts. Moreover, identifying mutations that are associated with phenotypic and clinical PZA^R^ may aid in the design of more sensitive and specific molecular assays for resistance detection. Among isolates collected from 25 States and four Union Territories in India, we sought to (i) estimate the prevalence of PZA^R^ and resistance-associated mutations, (ii) catalogue the diversity of known and potentially novel PZA^R^-associated mutations, and (iii) investigate the geographic distribution of the identified mutations. To meet these aims, whole genome sequencing (WGS) and pDST were completed on a national sample of MTB sputa and isolates prospectively collected from 2018 to 2020. To our knowledge, these findings represent the first, national, WGS-based characterization of pyrazinamide resistance in India.

## Materials and methods

### MTB clinical isolate selection and characterization

A total of 2,207 *M. tuberculosis-*complex (MTBC) sputum specimens and isolates were collected from intermediate and national reference laboratories across 25 Indian states and four Union Territories from 2018 to 2020. A quota sampling method was used for collection of samples, with state wise distribution based on 2017 DR TB burden estimates (Indian Council of Medical Research National Institute for Research in Tuberculosis, 2022). All samples had confirmed RIF and INH susceptibility patterns according to WHO-recommended molecular diagnostic and drug susceptibility tests (i.e., Cepheid Xpert MTB/RIF; Sunnyvale, CA, United States; Molbio Truenat MTB, Truenat MTB Plus, and Truenat Rif-Dx, Goa, India; and Bruker/Hain MTBDR*plus* and MTBDR*sl* assays, Nehren, Germany). The final collection was composed of 10% pan-susceptible, 10% mono-INH resistant, and 80% MDR (INH-and RIF-resistant) isolates and sputa, each representing a single case-patient.

### Phenotypic drug susceptibility testing

All samples referred by reference laboratories were cultured at the National Institute for Research in Tuberculosis (NIRT) on Löwenstein–Jensen (LJ) medium slants. Positive TB LJ cultures were sub-cultured using the BACTEC MGIT 960 system (BD, Franklin Lakes, NJ, United States). Positive MGIT cultures were used for MGIT-based pDST utilizing the 2018 WHO-recommended concentrations (World Health Organization, 2018) for 14 antitubercular drugs: INH (0.1 μg/mL), RIF (1.0 μg/mL), EMB (5.0 μg/mL), streptomycin (1.0 μg/mL), kanamycin (2.5 μg/mL), amikacin (1.0 μg/mL), capreomycin (2.5 μg/mL), ofloxacin (2.0 μg/mL), levofloxacin (1.5 μg/mL), moxifloxacin (0.5 μg/mL), para-amino salicylic acid (4.0 μg/mL), ethionamide (5.0 μg/mL), linezolid (1.5 μg/mL), and bedaquiline (0.5ug/mL). PZA DST was performed using MGIT 960 PZA kits at the recommended concentration of 100 μg/mL. The pan-susceptible MTB H37Rv strain was used for routine quality control. In case of discrepancy between phenotypic and genotypic DST results, repeat pDST was performed in duplicate to confirm phenotypic results. The replicate testing results were considered final for pDST. Isolates susceptible to all 14 drugs were categorized as pan-susceptible. Any isolate with resistance to one or more antituberculous drug was defined as drug resistant. Types of drug resistance were defined according to the resistance patterns outlined by World Health Organization (2022). For example, strains were defined as multidrug-resistant (MDR) if resistant to INH and RIF, as pre-XDR, if MDR with additional resistance to any fluoroquinolone, or as XDR if pre-XDR with additional resistance to bedaquiline or linezolid. Isolates with other resistance patterns were defined as mono-resistant to a single drug, or poly-resistant, if characterized by resistance patterns not otherwise covered by the definitions for MDR, pre-XDR, or XDR.

### DNA isolation

In addition to culture and phenotypic DST, all received samples underwent DNA isolation for downstream whole genome sequencing (WGS). Genomic DNA was extracted from LJ-amplified strains using the cetyltrimethylammonium bromide (CTAB) method and was purified by the Genomic DNA Clean and Concentrator kit (Zymo Research, Irvine, CA, United States). DNA quality and quantity were measured using NanoDrop 2000 spectrophotometer and Qubit dsDNA BR Assay kit (Thermo-Fisher Scientific, Waltham, MA, United States) following manufacturer’s instructions.

### Whole-genome sequencing

DNA libraries were prepared using NexteraXT DNA Library Preparation and Index kits (Illumina, San Diego, CA, United States). Average library sizes were measured ∼850 bp on the Bioanalyser 2,100 System (Agilent Technologies, Santa Clara, CA, United States), normalized in equimolar concentrations, and loaded for WGS (MiSeq Reagent Kit v3; Illumina, San Diego, CA, United States). The 2 × 251 cycles of paired end read sequencing were performed on a MiSeq sequencer (Illumina, San Diego, CA, United States). The raw sequence reads were deposited in NCBI with Bioproject (Accession ID: PRJNA1155695).

### Variant calling, lineage and drug resistance prediction

Sequence reads at least 60 bp minimum length and with base quality scores of 20 were filtered using Trimmomatic v0.36 (LEADING:20 TRAILING:20 SLIDINGWINDOW:4:20 MINLEN:60) (Bolger et al., 2014). Kraken v1.0 was used to identify potential contamination with other species (Wood and Salzberg, 2014). Kraken analysis provided the top species for each isolate along with relatedness percentages for any other species. Reads were mapped to the *M. tuberculosis* H37Rv reference genome (NC_000962.3) using bwa v0.7.12 with default parameters (Li, 2013). The coverage depth and breadth were calculated after alignment to the H37Rv reference genome. The isolates with >30x depth and > 85% breadth coverage to reference genome was included for downstream analysis. Indels mapping correction was done using Picard v2.2.4 and GATK v3.5. Samtools v1.3.1 and BCFtools v1.3.was used to identify variants with default (samtools mpileup-d 8,000-t DP-B -u-g -m 4 and bcftools call-m -v-o) (Li, 2013; Picard Toolkit, 2009; Danecek et al., 2021; McKenna et al., 2010). Variants were filtered based on the following metrics: base quality >50, mapping quality >30, read depth > 5 and at least one read mapping in either direction. Variants supported by >80% of the mapped reads were classified as homozygous sites and those with <80% mapped reads were classified as heterozygous sites. RD-analyzer was used to predict the lineages of isolates (Faksri et al., 2016). This tool predicts the lineage of a given isolate based on the presence or absence of 31 regions of difference (RD). Variants were compared against a database of mutations to predict published resistance-conferring mutations for first- and second-line antituberculosis drugs using a validated inhouse pipeline that was previously described elsewhere (Tamilzhalagan et al., 2021).

### Statistical analysis

Odds ratios (ORs) and corresponding 95% confidence intervals (CIs) were calculated to compare the effects of demographic characteristics, drug resistance profiles and lineage against PZA resistance. Sensitivity, specificity, positive predictive values (PPV), and negative predictive values (NPV) were calculated to assess the accuracy of gene and mutation associations with PZA^R^. To assess the association of novel mutations with resistance, we used the confidence grading method proposed by Miotto et al. (2017) which employs likelihood ratios (LR), ORs, and accompanying *p*-values to categorize mutations into high-confidence, moderate-confidence, minimal-confidence, indeterminate, and ‘no resistance’ associated mutations (Köser et al., 2020). In addition, any nonsense mutations, insertions, and deletions in the coding region of *pnc*A were classified as PZA^R^, in alignment with global resistance interpretation resources (World Health Organization, 2023). We utilized several software packages to conduct the various analyses (R, R Foundation for Statistical Computing, Vienna, Austria; MedCalc, Ostend, Belgium); R and Tableau were used to for graphic representations (Salesforce Inc., Mountainview, CA, United States) (R Core Team, 2021; Salesforce Inc., 2018; MedCalc, 2020).

## Results

### Phenotypic drug susceptibility testing outcomes

Of the 2,207 clinical isolates cultured, 944 (43%) were determined to be PZA^R^ and 1,263 (57%) were PZA susceptible (PZA^S^) by pDST. Initially, 476 (22%) isolates displayed discordant phenotype–genotype DST results and underwent repeat phenotypic testing. Subsequently, 117 isolates originally predicted to be PZA^R^ were recategorized as susceptible and 48 PZA^S^ isolates were recategorized as resistant, leaving 311 isolates with discordant DST results. After repeat testing, PZA resistance was most prevalent among XDR isolates (68%; 23/34), followed by those characterized as pre-XDR (64%; 756/1,178), MDR (31%; 133/432), and polydrug-resistant (13%; 29/230). Only 3 samples were found to be phenotypically PZA mono-resistant (Table 1).

**Table 1:** Detection of pyrazinamide resistance in India (2018–2020) based on phenotypic drug susceptibility testing and whole genome sequencing by TB drug resistance category.

Resistance category	Isolates, *n* (%)	WGS PZA resistant isolates, *n* (%)	pDST PZA resistant isolates, *n* (%)	gDST and pDST agreement, *n* (%)
Extensively drug-resistant (XDR)	34 (1.5)	21 (61.8)	23 (67.6)	17 (81.0)
Pre-extensively drug-resistant (pre-XDR)	1,178 (53.4)	660 (56.0)	756 (64.2)	609 (92.3)
Multidrug-resistant (MDR)	432 (19.6)	109 (25.2)	133 (30.8)	97 (89.0)
Polydrug-resistant^a^	230 (10.4)	6 (2.6)	29 (12.6)	4 (66.7)
PZA mono-resistant	5 (0.2)	5 (100)	3 (60.0)	3(60.0)
Pan-susceptible	328 (14.9)	–	–	–
Total	2,207 (100)	801/2,207 (36.3)	944/2,207 (42.8)	730/801 (91.1)^b^

Agreement between phenotypic and genotypic DSTs are shown in righthand column.

gDST, genotypic drug susceptibility testing; pDST, phenotypic drug susceptibility testing; MDR, multidrug-resistant tuberculosis (at least resistant to isoniazid and rifampin); pre-XDR, pre-extensively drug-resistant tuberculosis (MDR TB plus additional resistance to any fluoroquinolone); PZA, pyrazinamide; XDR, extensively drug-resistant tuberculosis (MDR TB plus resistance to any fluoroquinolone and bedaquiline or linezolid).

a Polydrug-resistant tuberculosis: resistance to two or more antituberculosis drugs and not classified MDR, pre-XDR or XDR.

b Total percent agreement between gDST and pDST for pyrazinamide was determined used genotypic resistance determination as a denominator because sequencing is the most likely gold standard for resistance determination of this antituberculosis drug.

### Whole genome sequencing-based pyrazinamide drug susceptibility profiles and their geographic distribution

Of the 2,207 isolates characterized, 328 (14.9%) were genotypically categorized as pan-susceptible, 5 (0.2%) as mono-PZA-resistant, 432 (19.6%) as MDR, 1,178 (53.4%) as pre-XDR, 34 (1.5%) as XDR, and 230 (10.4%) as polydrug-resistant (Table 1). A total of 801 (36%) were characterized as PZA-resistant by WGS. As observed with pDST results, the proportion of genotypic PZA^R^ was highest among XDR strains (62%), followed by those characterized as pre-XDR (56%), MDR (25%), polydrug-resistant (3%) and PZA mono-resistant (100%). Agreement between pDST and gDST for PZA^R^ determination was high overall (91%) and across resistance categories (60–92%) for isolates classified as resistant by gDST, which is emerging as the gold standard method for PZA drug susceptibility testing (Maningi et al., 2015; Rahman et al., 2017).

**Figure 1:**
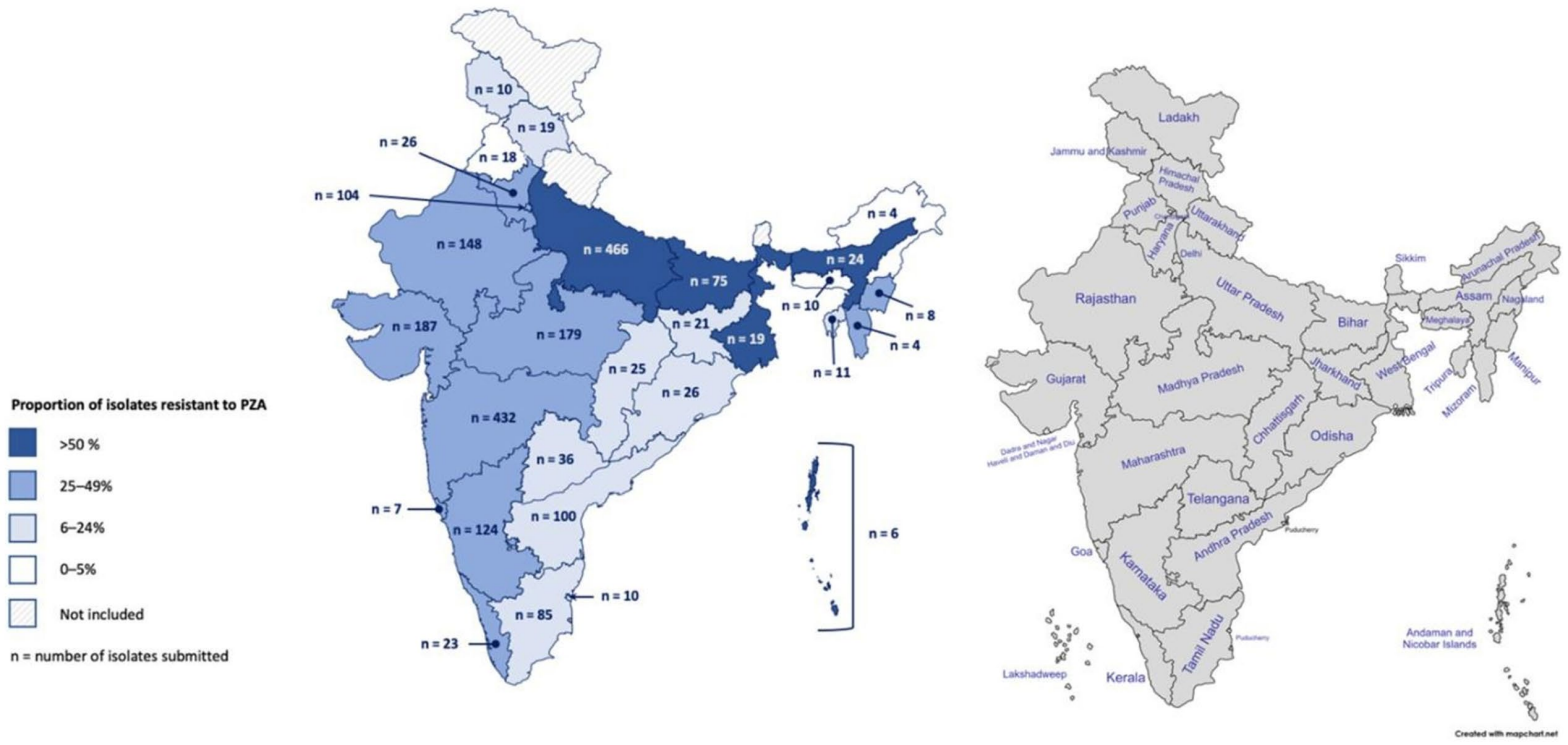
Geographic distribution of *Mycobacterium tuberculosis* isolates collected in India and the proportion resistant to pyrazinamide based on whole-genome sequencing. In total, 2,207 isolates were collected from 2018 to 2020 across 25 Indian states and 4 Union Territories.

Genotypic-based PZA^R^ was determined to be above the national prevalence in several geographically dispersed Indian states and Union Territories (Figure 1). While West Bengal, the Andaman and Nicobar Islands, Kerala, Bihar, and Assam had some of the highest proportions of PZA^R^ in this group (68, 50, 48, 47, and 46%, respectively), their results could be biased due to small sample sizes (19, 6, 23, 75, and 24, respectively). PZA^R^ prevalence in Uttar Pradesh (47%) and Maharashtra (46%), may be more robust, as each contributed >430 isolates of varying drug resistance patterns (Figure 1). The remaining submitting states and Union Territories with genotypically-detected PZA^R^ varied in isolate volume, resistance category diversity (data not shown), and proportion of PZA^R^ (9–38%). Punjab (*n* = 18) and Arunachal Pradesh (*n* = 4) were the only states that submitted isolates for which PZA^R^ was not detected.

### Factors associated with PZA resistance

Neither gender nor age were significantly associated with an increased risk of PZA^R^, as determined by WGS; however, PZA^R^ was significantly associated with resistance to all antitubercular drugs assessed (Table 2). The analysis showed the greatest association of PZA^R^ and EMB resistance (OR: 26.0; 95%CI: 17.8, 38.1), followed by cross-resistance to the first-line drugs RIF, INH, and EMB (OR: 23.6; 95%CI: 17.2, 32.3), streptomycin resistance (OR 18.4; 95%CI: 14.6, 23.2), fluoroquinolones (OR: 7.6; 95%CI: 6.1, 9.5), and para-amino salicylic acid (OR 6.3; 95%CI: 3.8, 10.7). While still elevated, odds of PZA^R^ were comparatively lower among isolates characterized as resistant to amikacin, ethionamide, and linezolid (Table 2). Overall, *M. tuberculosis* lineage was not significantly associated with increased odds of PZA^R^. However, a statistically higher proportion of Lineage 2 strains (64%) were PZA-resistant as compared to the proportions observed across all other lineages (17–34%), despite an overall predominance of Lineage 3 (Table 2).

**Table 2:** Demographics and strain-specific factors associated with pyrazinamide resistance in India (2018–2020), as determined by whole-genome sequencing.

Characteristic	Number of isolates	Genotypically PZA-resistant	Genotypically PZA-susceptible	Odds ratio	95% CI
	*N* = 2,207	*N* = 801	*N* = 1,406		
		*n* (%)			
Gender and age					
Male	1,325	454 (34.3)	871	Reference	
Female	694	267 (38.5)	427	0.8	0.7, 1.0
Not available	188	80 (42.6)	108	**–**	**–**
Age 0–17 years	255	102 (40.0)	153	1.2	1.0, 1.6
Age > 18 years	1735	619 (35.7)	1,116	–	–
Not available	217	80 (36.9)	137	–	–
Phenotypic drug resistance (MGIT)					
Ethambutol (5.0 μg/mL)	1,469	771 (52.5)	698	**26.0**	**17.8, 38.1**
Streptomycin (1.0 μg/mL)	1,155	647 (56.0)	508	**18.4**	**14.6, 23.2**
Amikacin (1.0 μg/mL)	223	130 (58.3)	93	**2.7**	**2.1, 3.6**
Ofloxacin (2.0 μg/mL)	1,291	683 (52.9)	608	**7.6**	**6.1, 9.5**
Levofloxacin (1.5 μg/mL)	1,291	683 (52.9)	608	**7.6**	**6.1, 9.5**
Moxifloxacin (0.5 μg/mL)	1,291	683 (52.9)	608	**7.6**	**6.1, 9.5**
Para-amino salicylic acid (4.0 μg/mL)	83	64 (77.1)	19	**6.3**	**3.8, 10.7**
Ethionamide (5.0 μg/mL)	397	231 (58.1)	166	**3.0**	**2.4, 3.9**
Linezolid (1.5 μg/mL)	36	21 (58.3)	15	**2.5**	**1.3, 4.8**
Any first-line drug (INH, RIF, EMB)	1,332	755 (56.7)	577	**23.6**	**17.2, 32.3**
Phylogenetic lineage					
Lineage 1 (East-Asian Indian)	357	62 (17.4)	295	0.3	**0.2, 0.4**
Lineage 2 (Beijing)	710	455 (64.1)	255	5.9	**4.9, 7.2**
Lineage 3 (Central Asian)	822	173 (21.1)	649	0.3	**0.3, 0.4**
Lineage 4 (Euro American)	275	94 (34.2)	181	0.9	0.7, 1.2
Mixed lineages	30	14 (46.7)	16	1.5	0.8, 3.2
H37*Rv*-like	13	3 (23.1)	10	0.5	0.1, 1.9

Characteristic-based odds ratios are presented and precision estimations are provided by 95% confidence intervals.

CI, confidence interval. Bold signifies statistical significance.

### *pnc*A mutations in isolates resistant to PZA by whole genome sequencing

**Figure 2:**
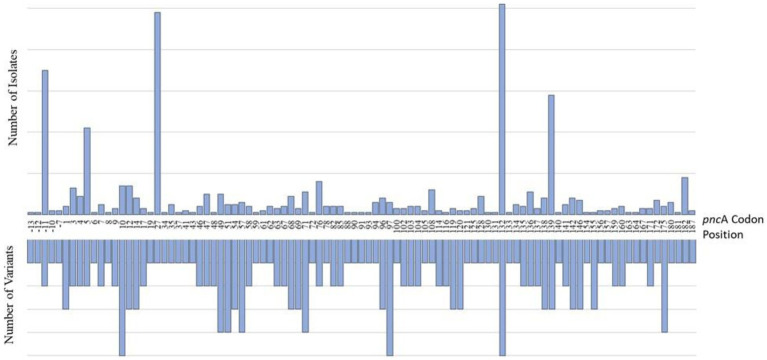
Characterization of *pnc*A mutations in PZA-resistant (PZA^R^) isolates determined by whole-genome sequencing. The number of unique resistance-conferring variants detected at codon positions across the *pnc*A promoter region and open reading frame are displayed on the lefthand side and the distribution of mutations identified in PZA^R^ isolates is shown on the righthand side.

Of the 801 isolates characterized as pyrazinamide-resistant by whole genome sequencing, 796 harbored mutations in the PZA^R^-associated gene, *pnc*A. These included 77% of all phenotypically PZA-resistant isolates (728/944) and 99% of all genotypically-resistant isolates (796/801). The number of isolates harboring at least one *pnc*A mutation at any coding position across the entire gene is shown in Figure 2. As expected, most genotypic resistant isolates (99%) harbored mutations throughout the *pnc*A open reading frame, with those harboring mutations at codon positions 132, 27, 139, and 5 predominating among 102, 98, 58, and 42 isolates, respectively. An additional 76 isolates were found to have promoter region mutations, the majority (*n* = 70) of which carried mutations at the-11 codon position. As expected, 216 (23%) of the phenotypically PZA-resistant isolates had no known *pnc*A resistance markers.

The frequency and distribution of *pnc*A mutations, and the number of unique variants by codon position are shown in Figure 2. Investigation of the entire *pnc*A gene, revealed 171 distinct resistance-associated mutations (Supplementary Table S1). The promoter region harbored six (4%) mutations while the remaining 165 (96%) were distributed throughout the *pnc*A open reading frame. Nonsynonymous, single nucleotide polymorphisms (SNPs) causing amino acid substitutions were predominant (*n* = 170) alongside a single guanine deletion at nucleotide position 342. Of the 187 codons investigated (with the exception of the guanine deletion), 95 (51%) harbored at least one variant, whereas the remaining 92 (49%) were not associated with mutations. Variation was most notable at *pnc*A positions 10 (*n* = 5), 97 (*n* = 5), 132 (*n* = 5) with five variants each, followed by four variants in each of the codons at positions 49, 51, 57, 71 and 175 (Figure 2).

The most frequent PZA^R^ mutation, the Lineage 2-specific amino acid change L27P, was associated with PZA^R^ in 97 isolates, which represented 12% of all genotypically-PZA-resistant isolates. The next most frequent mutation was G132A, detected in 96 isolates, 90 of which also belonged to Lineage 2. Seventeen isolates were found to contain multiple PZA-resistance associated mutations in *pnc*A, including 13 double mutants and four triple mutants.

### *pnc*A mutations in isolates phenotypically susceptible to PZA

The 1,263 PZA^S^ isolates by culture-based drug susceptibility testing were found to harbor 39 *pnc*A mutations predicted to confer resistance based on our in-house pipeline. Among these, 37 (95%) were within the coding region and two (5%) were located in the promoter region (Supplementary Table S1). Twenty-nine of these mutations were also associated with phenotypic PZA^R^ in at least two MTB isolates. However, nine mutations genotypically associated with resistance (M1K, D63A, D63G, P69L, H137R, V139M, Q141STOP, V155L, E181D) were only identified in 68 (5%) phenotypically susceptible isolates.

### Lineage analysis of *pnc*A mutations

Despite a known predominance of Lineage 3 in India, the proportion of WGS-based PZA^R^ isolates was only 21% (173/822), with approximately three times as many PZA^R^ isolates (64%; 455/710) belonging to Lineage 2. In total, Lineage 2 constituted 57% of those determined to be genotypically resistant, followed by Lineage 3 (22%), and then Lineage 4 (12%) and Lineage 1 (8%). It is noteworthy that 4 of 5 (80%) PZA mono-resistant isolates belong to Lineage 1. We observed that PZA^R^ in Goa, Andaman and Nicobar, Jharkhand, Chattisgarh, Meghalaya, Mizoram, Nagaland, Odisha was confined to Lineage 2 isolates, regardless of the presence of multiple lineages found in those regions. In addition, eight lineage-specific resistance makers were detected. The mutations L27P (*n* = 97), L182S (*n* = 18), A3E (*n* = 10), H71Y (*n* = 8), G78V (*n* = 4), T142A (*n* = 4) were identified only in Lineage 2, while mutations F94L (*n* = 6) and V7G (*n* = 4) were specific to Lineage 3 and Lineage 4, respectively. Four coding mutations S67P, D12A, L172P and V139A were observed across all four lineages.

A lineage-wise comparative analysis of mutations associated with PZA^R^ across the *pnc*A promoter and coding region was conducted. While slightly higher proportions (≥30%) of PZA^R^-associated mutations were observed for Lineage 2 isolates between codon positions 1–30 (35%; 159/460) and 121–150 (34%; 158/460) and for Lineage 1 isolates between codon positions 61–90 (30%; 19/63), no lineage-specific “*hotspots*” were identified in *pnc*A. Compared to other Lineages, Lineage 2 isolates were shown to harbor a wider spectrum of mutations across more codon positions (data not shown), but overall, mutations were distributed broadly across the *pnc*A regulator region and open reading frame and revealed overlapping patterns between lineages.

### Mutations in *rps*A, *pan*D and *clp*C1 genes

Although *rps*A and *pan*D have been reported to be potential targets for PZA resistance, no substantial number of mutations associated with resistance were observed in either of these genes (Table 3). A single non-synonymous mutation in *rps*A, E67D, was identified in a phenotypic PZA^S^ isolate and a synonymous lineage marker R212R in *rps*A was found in all Lineage 2 isolates. Investigation of PZA^R^ isolates without *pnc*A mutations revealed two of 216 contained the resistance-conferring mutation I49V in *pan*D. However, this mutation was also detected in two other isolates determined to be susceptible to PZA. Analysis of *clp*C1 revealed the presence of a coding mutation V63A in 323 isolates, all belonging to Lineage 1, 68 of which were determined to be PZA^R^ owing to the co-occurrence of *pnc*A mutations. Isolates with V63A mutation devoid of *pnc*A mutations were phenotypically PZA^S^ implying their lack of association with resistance at the tested critical concentration. The synonymous mutation N806N in *clp*C1 was detected in 37 isolates (11 PZA^R^, 26 PZA^S^), all from Lineage 4. A single, novel mutation upstream of *clp*C1 (A-15G) in the promoter region was detected in eight phenotypic-based PZA^R^ isolates and six PZA^S^ isolates, indicating moderate resistance association, as determined by confidence grading.

**Table 3:** Mutations in *rps*A*, pan*D and *clp*C1 genes associated PZA-resistant and PZA-susceptible isolates in India (2018–2020).

Nucleotide position based on the MTB H37*Rv* reference genome		Codon change	Amino acid change	Codon position	Number of isolates
PZA-resistant isolates					
*pan*D	4,044,137	ATC/GTC	I → V	49	2
*clp*C1	4,040,517	GTC/GCC	V → A	63	68
	4,038,287	AAC/AAT	N → N	806	11
PZA-susceptible isolates					
*pan*D	4,044,137	ATC/GTC	I → V	49	2
*rps*A	1,833,742	GAA/GAC	E → D	67	1
*clp*C1	4,040,517	GTC/GCC	V → A	63	255
	4,038,287	AAC/AAT	N → N	806	26

Codon and amino acid changes are listed along with positions in each respective gene.

A, alanine; D, aspartic acid; E, glutamic acid; I, isoleucine; N, asparagine; PZA, pyrazinamide; V, valine.

### Novel mutations associated with PZA resistance

To identify novel mutations associated with PZA^R^, initially we extracted previously reported mutations exclusive to isolates characterized as resistant by both WGS and pDST. However, each mutation identified was only found in one isolate so the ability to determine associations with resistance was not possible. Thus, as an alternative approach, we extracted mutations in any gene which were present in 214 phenotypically PZA^R^ and genotypically PZA^S^ discordant isolates and compared those mutations found in 1,192 phenotypic and genotypically PZA^S^ isolates. As a result, 16,848 mutations were found; 243 were predicted to have a high-confidence association with resistance, and 166 a moderate association with resistance, based on the confidence grading method by Miotto et al. (2017). In total, 409 group A mutations were identified based on the newer categorisation method by Köser et al. (2020), including four *pnc*A high-confidence mutations (T177P, V7A, ACC392ACCCC, CG394CGG) (Supplementary Table S2). Based on confidence grading, several additional genes were identified that contained mutations with high-confidence association to PZA^R^. A203P in Rv2492, G221D in Rv0939, Y206S in PPE1 (Rv0096), A-17G in *rpf*B (Rv1009) and D595D in Rv0102 were the most common. For Tier 2 candidate genes associated with resistance to PZA (World Health Organization, 2023), two mutations with a weak association to phenotypic resistance (minimal confidence) were identified, one in Rv1258c and the other in PPE35 (Rv1918c).

Based on expert rules outlined in the WHO catalogue of mutations that any nonsense mutation and insertions/deletions (indels) in the coding region of *pnc*A, as well as all non-synonymous mutations presumed to cause loss of function resistance phenotypes (unless disproven) if occurring in the coding region of *pnc*A, we identified 143 additional mutations meeting this criterion. These mutations were found in 159 of 214 isolates with discordant phenotypic and genotypic DST results and included numerous indels varying in nucleotide length.

### Accuracy of WGS-based prediction of PZA resistance

The correlation between WGS data and phenotypic data was established for all 2,207 isolates. After replicate pDST was performed for initial discordant isolates, overall concordance was observed for 1,922 (87%) isolates. Using phenotypic results as the reference standard, based on detection of the 171 mutations in *pnc*A known to confer resistance, sequencing exhibited a sensitivity of 77% (95% CI: 74.4, 79.8) and a specificity of 94.6% (95% CI: 93.3, 95.9) (Table 4). Considering the additional mutations associated with resistance based on inclusion of the expert rule (*n* = 143) as well as the 171 mutations known to confer resistance, the sensitivity of *pnc*A sequencing to accurately detect resistance substantially increased to 94%; however, the specificity decreased to 90%. Unfortunately, the calculated sensitivity and specificity *pan*D were not reliable due to small size (2 isolates) (Bujang and Adnan, 2016).

**Table 4:** Accuracy of whole genome sequencing *pnc*A and *pan*D genes to predict PZA resistance in India (2018–2020).

Gene(s)	Mutations^a^ in PZA-resistant isolates (*N* = 944)	Mutations^a^ in PZA-susceptible isolates (*N* = 1,263)	Sensitivity	Specificity
	*n* (%)	*n* (%)	(95% CI)	(95% CI)
*pnc*A (No expert rule)	728 (77.1%)	68 (5.3%)	77.1	94.6
			(74.4, 79.8)	(93.4, 95.9)
*pnc*A^b^ (Expert rule)	887 (94.0%)	123 (9.7%)	94.0	90.3
			(92.3, 95.4)	(88.5, 91.8)
*pan*D^c^	2 (0.0%)	2 (0.0%)	0.2	99.8
			(0.0, 0.5)	(99.6, 100)
*pan*D *and pnc*A	730 (77.3%)	70 (5.5%)	77.3	94.5
			(74.7, 80.0)	(93.2, 95.7)

Sensitivities and specificities, including 95% confidence intervals, were calculated to determine accuracy of sequencing *pnc*A (with and without application of expert rules), *pan*D, and the combination of sequencing *pnc*A and *pan*D.

a Known mutations include both nonsynonymous mutations, insertions, and deletions.

b *pnc*A analysis for mutations associated with PZA resistance including application of the expert rule that defines any loss of function *pnc*A mutation, increasing the number of resistance-associated mutations (*n* = 143).

c Calculated sensitivity and specificity are not reliable due to small size (Bujang and Adnan, 2016).

## Discussion

PZA is a first-line anti-TB drug and can play an important role in the clinical outcomes of TB patients. However, challenges associated with phenotypic, culture-based DST such as long turnaround times and unreliable results can lead to delays in treatment onset or misdiagnosis of PZA^R^. Molecular-based DST can provide faster and more accurate prediction of PZA^R^, in particular NGS has been shown to improve detection when used as the gold standard method, can help settle discordant results obtained from conventional DSTs, and has proven to be a valuable tool for surveillance of drug resistance (Rajendran and Palaniyandi, 2022; Maningi et al., 2015). In India, where the incidence of MDR TB is high, reliable prediction of PZA^R^ can support judicious use of this key drug and thwart onward transmission of resistance. As limited studies have been conducted in high DR TB burden countries to characterize PZA resistance, this nationwide investigation of 2,207 MTB isolates aimed to explore the spectrum of mutations that confer resistance to PZA, assess the current prevalence of PZA^R^ in India, and most importantly, evaluate the diagnostic potential of sequencing *pnc*A, *rps*A, *pan*D and *clp*C1 genes after assessing their roles in PZA^R^.

Based on data collected from 2015 to 2022, the estimated pooled prevalence of phenotypic PZA^R^ among MDR TB patients in the WHO South-East Asian Region (SEAR) was 37% (Wang et al., 2023). Data from this study conducted from 2018 to 2020 indicates the prevalence of PZA^R^ within this population in India was slightly lower at 30.8%. Prevalence in India was also lower than other countries in SEAR including Bangladesh (45%) (Rahman et al., 2017), and Myanmar (59%) (Ei et al., 2020). Compared to India, considerably higher prevalence of PZA^R^ in MDR TB cases was also reported in Sub-Saharan Africa (54%) (Ngabonziza et al., 2017), South Africa and the Republic of Georgia (56–66%) (Allana et al., 2017), and Southern China (69%) (Pang et al., 2017). However, our current estimate in India aligns with the previous national prevalence of PZA resistance estimated to be 31% among persons with MDR TB (Government of India Ministry of Health and Family Welfare, 2018). Geographically, genotypic-based PZA^R^ was determined to be above the national prevalence in several Indian states and Union Territories including in Maharashtra (46%), reported to have the highest prevalence of MDR in a state-wise analysis (Lohiya et al., 2020) and Uttar Pradesh (47%), which as of 2018 accounted for approximately 20% of the rifampicin-resistant and MDR TB cases in the country (Government of India Ministry of Health and Family Welfare, 2018).

In line with the observation made by Ramirez-Busby and Valafar (2015), broader resistance directly translated to greater prevalence of PZA^R^ in our analysis. More extensive resistance to anti-tubercular agents resulted in a higher proportion of PZA^R^. Our correlative analysis also highlighted the significant relationship between genotypic PZA^R^ and resistance to 13 other antituberculosis drugs. This is consistent with several studies which have reported concomitant PZA^R^ with resistance to other first-line drugs and fluoroquinolones (Whitfield et al., 2015; Alame-Emane et al., 2015; Shi et al., 2020; Whitfield et al., 2016). PZA co-resistance was highest in isolates resistant to ethambutol, followed by any first-line drugs (INH, RIF and EMB) and streptomycin. PZA co-resistance was comparatively less likely in isolates with resistance to amikacin, ethionamide, and linezolid. The notable association of PZA^R^ to 13 drugs is concerning and signals the imminent need to scale-up accurate PZA^R^ determination and confirm PZA susceptibility prior to use in all regimens to arrest resistance amplification.

Differences in genetic backgrounds of *M. tuberculosis* strains may effect the transmissibility of drug-resistant tuberculosis and it has been shown that certain lineages have been linked with specific resistance-associated mutations and phenotypic drug resistance (Fenner et al., 2012). The tendency to acquire resistance mutations is not equal between MTB lineages and the strong association of drug resistance with Lineage 2 isolates has been demonstrated across different countries (Borrell and Gagneux, 2009; Ford et al., 2013). Within our dataset, despite a higher number of Lineage 3 isolates, isolates belonging to Lineage 2 showed approximately three times the proportion of PZA^R^ as well as a wider spectrum of *pnc*A mutations. However, contrary to results reported by Baddam et al. (2018) notable overlap of mutations was documented in this work between isolates of different lineages at any position. While slightly higher proportions of resistance determining mutations were detected in certain regions of the *pnc*A gene, no ‘*hot spots*’ were identified. Similarly observed for global datasets (Whitfield et al., 2015), given the diversity of mutations and noting that high-confidence resistance-conferring variants were distributed throughout *pnc*A and its associated promoter region among these Indian isolates, development of targeted molecular DSTs proves challenging and highlights the importance of full-length gene sequencing for more accurate prediction of resistance.

In *M. tuberculosis*, homoplasy is evident due to natural selection of resistance determining mutations by way of convergent evolution (Outhred et al., 2020). Herein, six of 171 PZA resistance-associated mutations (A-11G, A146T, S67P, D12A, L172P and V139A) were found in all four lineages, indicating homoplasy and a high likelihood of natural selection. The number of mutations identified in three separate lineages (*n* = 19) was half as many identified in two lineages (*n* = 38). Investigation of mutations specific to each lineage was conducted to analyze their impact on PZA^R^ in India. Several lineage-specific mutations were identified within this dataset, including the most common, Lineage 2-specific, non-synonymous L27P mutation in the *pnc*A open reading frame, along with L182S, A3E, H71Y, G78V, T142A. Similarly, we observed one mutation to be specific to Lineage 3 (F94L) and another mutation specific to Lineage 4 (V7G). Four out of five (80%) PZA mono-resistant isolates belonged to Lineage 1, similarly observed by Modlin et al. (2021) and Mok et al. (2021) in which isolates belonging to this lineage were overrepresented in PZA-mono-resistant TB.

Along with *pnc*A, The WHO Mutations Catalogue lists *clp*C1 and *pan*D as Tier 1 candidate genes associated with resistance to PZA (World Health Organization, 2023). The *clp*C1 V63A mutation is considered a phylogenetic variant associated with the East Africa Indian (EAI) sublineage, coinciding with our findings this mutation was detected solely in Lineage 1 isolates. At first glance, our data suggests this mutation lacks association with resistance as only isolates with concurrent *pnc*A mutations were found to be phenotypically PZA^R^. However, Mok et al. (2021) describe PZA^R^ isolates harboring only a V63A mutation (*pnc*A wild-type), but their results indicate EAI isolates display an elevated background MIC when using a reduced inoculum method. As V63A mutations have been suggested to be associated with low-level PZA resistance (Modlin et al., 2021), it is possible lower resistance levels may be missed in this work based on use of the single recommended PZA concentration (100 μg/mL). In addition, one novel mutation in the promoter region of *clp*C1 (A-15G) was detected in PZA^R^ (*n* = 8) and PZA^s^ (*n* = 6 isolates) resulting in a moderate link to resistance. Supplementary research is needed to determine the range of PZA resistance and clinical significance of isolates carrying the V63A mutation as well as to better understand the novel *clp*C1 promoter mutation, in the Indian context and globally.

Reports have documented PZA^R^ MTB isolates harboring *pan*D mutations, including I49V, but devoid of mutations in *pnc*A (Zhang et al., 2013; Werngren et al., 2017). In this Indian dataset, I49V was found in four Lineage 1 isolates, two of which were determined to be resistant and two susceptible by pDST. The absence of *pnc*A mutations in the phenotypically resistant isolates containing only a *pan*D mutation demonstrates the independence the latter gene’s ability to confer resistance or the possibility that other uncharacterized mechanisms of PZA resistance may exist. Conflicting evidence has both supported and dismissed the role of *rps*A in PZA resistance, but similar to Alexander et al. (2012), sequencing *rps*A in Indian isolates had no additional yield for predicting PZA^R^ and no mutations associated with resistance were detected. Overall, given the limited incidence and lack of high-confidence PZA-resistance associated mutations in *rps*A and *pan*D mutations in this Indian dataset, inclusion of these targets into molecular diagnostic approaches regionally may not significantly improve the accuracy of detecting resistance. However, evolution of molecular DSTs may be needed in other regions with higher of incidence of *pnc*A-independent mutations known to confer resistance or if future studies in India discover increasing frequencies of high-confidence *rps*A or *pan*D mutations associated with PZA^R^.

Studies have shown that culture-based susceptibility testing for PZA can be difficult to perform, resulting in an overestimation of resistance due to a variety of challenges associated with the test (Chang et al., 2011; Piersimoni et al., 2013). As part of this work, repeat pDST reduced the percentage of discordant results (pDST vs. WGS) from 22% down to 14%, demonstrating the difficulties associated with the gold standard method. However, repeat pDST still indicated a false positivity rate of ~1%. A small proportion (13/71, 18%,) of MTB isolates determined to be susceptible by pDST, were found to be resistant by WGS due to heteroresistance and oddly one isolate harboring two high-confidence PZA resistance-associated mutations (H71Y and Q10P) was found to be susceptible by pDST, during initial and repeat testing, indicating a potential co-occurrent affect. One study limitation includes at the time these analyses were conducted, calculations of discordance between phenotypic and genotypic-based methods were based on analysis of WGS data using our in-house bioinformatics pipeline; dependent on a database of published mutations known to confer PZA^R^ and confidence gradings outlined by Miotto et al. (2017). With the release of the WHO mutation catalogues in 2021, and most recently version two in 2023, application of additional, endorsed grading rules (expert rules), including loss of function mutations in *pnc*A, was shown to improve combined sensitivity for predicting phenotypic PZA resistance (World Health Organization, 2023). To overcome this initial limitation, we subsequently applied this additional grading rule to our Indian dataset, which improved sensitivity of WGS-based prediction of PZA^R^ from 77 to 94%.

A few other study limitations exist including enzymatic PZAse assays were not performed to confirm phenotypic DST results orthogonally, so it is unknown if all PZA^S^ MTB isolates displayed PZAse activity. In addition, assessing a range of PZA concentrations to determine MICs of phenotypically susceptible MTB isolates may have uncovered instances of low-level resistance and helped settle discordance between isolates harboring resistance-conferring mutations. Unfortunately, these findings were not correlated with treatment outcomes, which were not available during the time of data collection and analysis.

To the best of our knowledge, these findings represent the first, national, WGS-based characterization of pyrazinamide resistance in India. Despite limitations outlined, this study successfully explored the prevalence of mutations conferring PZA resistance, catalogued mutation diversity, investigated lineage specific associations of PZA resistance, examined co-resistance to first-and second-line drugs, and evaluated the diagnostic accuracy of sequencing for PZA resistance prediction. *pnc*A mutations across *pnc*A have been classified as markers of PZA resistance with high-confidence and sequencing the entire *pnc*A gene has been demonstrated as a reliable, accurate method for predicting phenotypic PZA^R^. A recent multi-national assessment of *pnc*A sequencing from 10,209 MTB isolates showed a good sensitivity and specificity of 92 and 97%, respectively (CRyPTIC Consortium and the 100,000 Genomes Project et al., 2018). Similarly, in India we demonstrated the diagnostic benefit of using sequencing to predict PZA resistance with sensitivity of 94% (95% CI: 92.3, 95.4) and specificity of 90% (95% CI: 88.5, 91.8), offering a reliable alternative to the technically-challenging and time-consuming gold-standard phenotypic method.

An understanding of regional differences in the sensitivity and specificity of sequencing-based DSTs underscores the importance of identifying circulating mutations known to confer resistance to PZA to tailor molecular diagnostics accordingly. Future characterization of currently unknown mechanisms conferring PZA resistance, addition of mutations with lower allelic frequencies to databases, better understanding of mutations associated with low-level resistance, and continued efforts collecting matching pDST and WGS data for MTB isolates to shed light resistance associations will improve the performance of genotypic-based prediction of phenotypic PZA resistance, regionally and globally. Overall, this work provides a first account and important insight into the scope of PZA-resistance in India, a high drug-resistant TB burden country and can support TB prevention and control efforts.

## Data Availability

Raw sequence reads were deposited in an NCBI Bioproject (accession ID: PRJNA1155695). All the samples used in this study are given in Supplementary Table S3.

## Supplementary material

SUPPLEMENTARY TABLE S1 PZA resistance-conferring mutations identified among phenotypically resistant and susceptible *M. tuberculosis* isolates. Nucleotide positions, codon changes, amino acid changes, and number of resistant and susceptible isolates containing each mutation (*n*=171) are listed.

SUPPLEMENTARY TABLE S2 Novel mutations associated with PZA resistance across the *M. tuberculosis* genome. Based on confidence grading, mutations were categorized into four groups, and mutations with high and intermediate associations with PZA resistance are presented (*n*=16,848). Genes, codon changes, amino acid changes, sensitivities, specificities, odds ratios, likelihood ratios, associated P-values are also presented for each mutation.

SUPPLEMENTARY TABLE S3 Project Accession, Sample Name, Biosample Accession, SRA Accession and Submission IDs of 2,207 samples. A total of 2,226 whole genome sequences (WGS) have been uploaded to GenBank; however, only 2,207 were utilized for this publication. The specific samples included in this study are listed in this table.
